# Animal Nutrition and Lipids in Animal Products and Their Contribution to Human Intake and Health

**DOI:** 10.3390/nu1010071

**Published:** 2009-08-14

**Authors:** Ian Givens

**Affiliations:** Animal Sciences Research Group, School of Agriculture, Policy and Development, Faculty of Life Sciences, University of Reading, Reading RG6 6AR, UK; Email: d.i.givens@reading.ac.uk.

**Keywords:** lipids, animal nutrition

## Abstract

Few EU countries meet targets for saturated fatty acid (SFA) intake. Dairy products usually represent the single largest source of SFA, yet evidence indicates that milk has cardioprotective properties. Options for replacing some of the SFA in milk fat with *cis*-monounsaturated fatty acids (MUFA) through alteration of the cow’s diet are examined. Also, few people achieve minimum recommended intakes (~450–500 mg/d) of the long chain n-3 polyunsaturated fatty acids (PUFA) eicosapentaenoic acid (EPA) and docosahexaenoic acid (DHA). Enrichment of EPA+DHA in poultry meat via bird nutrition is described and how this would impact on habitual intake is discussed.

## 1. Introduction

Public health nutrition is facing multiple and major challenges. There are in particular two major forces at work, the rapidly increasing burden of obesity and the increasing age of populations. Both trends increase the risk of chronic disease substantially and mean that diet, a key moderator of risk will play an increasingly important role. Data from WHO/FAO [1] suggest that by 2020 chronic diseases will account for almost 75% of all deaths worldwide with the vast majority being attributable to cardiovascular disease (CVD) with the associated rapid increase in the obesity/type 2 diabetes syndrome being particularly worrying as it is now also beginning to affect younger people. In the UK, the recent Foresight Report on obesity predicts that by 2050 some 55% of UK adults will be obese [2]. Similarly projections to 2050 of the age structure of the EU 25 suggests that its old-age dependency ratio (the number of people 65 and over relative to those between 15 and 64) is projected to double to 54% by 2050 [3]. The outcome of these trends will, if not moderated, lead to unsustainable costs of health care. Already, CVD costs the EU some €200 billion/year in direct and indirect charges [4].

It has been known for many years that diet plays a key role as a risk factor for chronic disease. In particular the effect of dietary fat in terms of both amount and type on the risk of chronic disease had much attention. Most attention has been focused on CVD though now it is recognised that effects of dietary fatty acids can be more widespread and subtle including effects on insulin sensitivity [5] and brain function [6]. Animal-derived foods contribute a substantial part of dietary fat in many countries and this has often been believed to be associated with increased chronic disease. This paper will focus on issues concerning intake of SFA and the long chain n-3 fatty acids (EPA and DHA) and examine opportunities to modify the composition of animal fats through animal nutrition in ways which may bring benefits in relation to long term health.

## 2. Saturated Fatty Acids and Chronic Disease

There is consistent evidence that dietary SFA increase the concentrations of serum LDL- cholesterol (LDL-C), an identified predictor of CVD risk and of coronary heart disease (CHD) in particular [7]. Until recently most attention has focused on LDL-C. However a meta-analysis of 60 selected human studies [8] confirmed that when dietary carbohydrates are substituted by an isoenergetic amount of C12:0 to C16:0 SFA, an increase in LDL-cholesterol does occur, but crucially the study also showed that there was a concomitant increase in the protective HDL-cholesterol (HDL-C). These workers argued that the ratio of total cholesterol to HDL-C provides the best predictor of the effect of dietary fatty acids on the risk of CHD. This interpretation indicates that the effects of C12:0 and C14:0 fatty acids may be rather beneficial as they both lower the total to HDL-C ratio whilst the opposite is the case for C16:0. The meta-analysis of Mensink *et al*. [8] also showed that overall, the risk of CHD would be most effectively reduced by the replacement of dietary SFA with either *cis*-MUFA or PUFA. The benefits of the two replacement strategies in terms of the resulting serum cholesterol profile, was similar. 

The replacement of SFA by either *cis*-MUFA or PUFA may have other beneficial outcomes. As noted above there is now some evidence that high intake of SFA may also be associated with reduced insulin sensitivity, a key factor in the development of the metabolic syndrome [9]. There is epidemiological evidence to support the association between high SFA intake and impaired glucose tolerance [10,11,12] and some intervention evidence. In a study involving 162 healthy subjects [5] given diets rich in SFA (from butter and margarine) or *cis*-MUFA (from high oleic sunflower oil) those on the SFA diet had significantly impaired insulin sensitivity (–10%) whilst those on the cis-MUFA diet showed no change. It is noteworthy however that the favourable effects of *cis*-MUFA were not seen in subjects with a high fat intake (>37% of energy intake). The same moderating effect of dietary fat intake has also been reported recently from the Lipgene study [13]. 

Despite the well publicised benefits of limiting SFA intake, populations in many parts of the EU still fail to meet dietary targets. Dietary targets vary from <11% of energy intake (EI) in the UK (population mean), <10% of EI [1] to as low as <7% of EI as recommended by the American Heart Association [14]. Table 1 shows SFA intake in various EU countries based on the TRANSFAIR study [15] and indicates that only Italy, Portugal, Spain and Greece achieve the target of <11% of EI. In the UK it was reported that about 78 and 74 % 0f men and women respectively exceeded the mean population target (<11% EI) [16]. Of great concern in the UK is the fact that children exceed the target by an even greater degree [17]. There does seem therefore considerable scope for reduction in SFA intake. 

**Table 1:** Intakes of saturated fatty acids (SFA) and the proportion supplied by dairy products in 11 EU Member States (adapted from [15]).

Country	Male (m) Female (f)	SFA intake (g/d)	SFA intake (% energy intake)	SFA from dairy (% total)
Belgium	m	45.0	15.3	30.2
	f	35.9	15.5	30.2
Finland	m	32.9	12.5	44.9
	f	25.2	12.8	44.9
France	m	34.2	15.0	56.7
	f	25.9	15.8	56.7
Germany	m	49.0	17.5	57.1
	f	39.2	18.6	57.1
Greece	m	22.6	10.5	27.4
	f	24.1	12.9	27.4
Italy	m+f	30.9	10.6	47.3
Netherlands	m	40.0	13.9	33.9
	f	30.7	14.3	33.9
Portugal	m	28.1	11.2	32.5
Spain	m+f	33.5	11.7	27.5
Sweden	m	37.7	15.0	48.5
	f	27.8	14.5	48.5
UK	m+f	27.1	13.2	38.8
Overall mean		33.5	14.1	41.3

### 2.1. Milk and Dairy Products as Sources of Saturates

Based on the TRANSFAIR study [15] Table 1 also shows the contribution to SFA intake made by milk and dairy products. The study also confirmed that on average milk fat contributed about 41% of all SFA and often contributes the single largest source of SFA. Thus logically, reducing the intake of SFA would simply require a reduction in consumption of milk and dairy products. However it is recognised that these foods are also key sources of other nutrients such as calcium and vitamin B_12_ [18]. 

Moreover there is now convincing epidemiological evidence that high intake of milk can provide long term reductions in the risk of CVD. Recently, from a meta-analysis of 15 studies the relative risk of stroke and heart disease in subjects with high milk or dairy consumption was shown to be 0.79 (95% CI 0.75, 0.82) and 0.84 (95% CI 0.76, 0.93) respectively, relative to the risk in those with low consumption [19]. Four studies reported incident diabetes as an outcome, and the relative risk in the subjects with the highest intake of milk or diary foods was 0.92 (95% CI 0.86, 0.97). Set against the proportion of total deaths attributable to the life-threatening diseases in the UK, vascular disease, diabetes and cancer, Elwood *et al*. [19] concluded that the results provide evidence of an overall survival advantage from the consumption of milk. Thus simply reducing milk/dairy consumption in order to reduce SFA intake is not likely to produce benefits overall. It should be noted however that the epidemiological evidence relates mainly to milk and in the UK at least, cheese and butter provide most of the dairy-derived SFA [16].

Based on the above evidence it would seem that changing the fatty acid composition of dairy foods to replace some SFA with *cis*-MUFA could provide a useful way to reduce SFA intake whilst retaining the cardioprotective benefits of milk. This possibility has recently been reviewed [20]. Based on the rather limited number of intervention studies with milk/milk products with SFA reduced from typically 70 to 55% of total fatty acids and *cis*-MUFA increased from typically 20 to 33% of total fatty acids, the review concluded that there was considerable potential for lower SFA dairy products to reduce population SFA intakes and potentially CHD burden. As part of a modelling exercise it was predicted that in the EU 27 reductions of some 10,500 and 3,900 deaths from CHD and stroke *per annum*, respectively, could result from such a strategy [21]. 

### 2.2. Modifying Milk Fatty Acid Composition

Milk fat typically contains 70-75 % SFA, 20-25% MUFA and small (2–5%) amounts of PUFA. Fatty acids in milk fat originate from two sources, either by direct incorporation from the peripheral circulation or from *de novo* synthesis in the mammary gland using short chain (C2:0 and C4:0) precursors. Mammary *de novo* synthesis accounts for all C4:0 to C12:0 fatty acids, most of the C14:0 and typically about half of C16:0 secreted in milk, while all C18 and longer chain fatty acids are derived entirely from circulating plasma lipids [22]. Due to extensive biohydrogenation of dietary unsaturated fatty acids by bacteria in the rumen, C18:0 is, under most conditions, the predominant long chain fatty acid available for absorption but the activity of stearoyl Co A (Δ-9) desaturase in mammary secretory cells converts a considerable amount of C18:0 to *cis*-9 C18:1 [23]. As noted above, the production of reduced-SFA dairy products could potentially be of large public health benefit. This can be achieved by increasing the supply of long chain fatty acids (of chain length C18 and above) to the mammary gland which inhibits the synthesis of short and medium chain saturates [24]. Thus use of oil seeds in cows’ diets typically produces reductions in the SFA content of milk fat from 70 to 50% (see review [25]). A recent study [26] examined the effect of form of rapeseed lipid in the diet of the dairy cow on the reduction in SFA and increase in *cis*-MUFA that could be achieved. The treatments were no rapeseed lipid (control) or rape lipid in the form of rapeseed oil, whole, unprocessed rapeseeds or milled rapeseeds. Some key results are summarised in Table 2. It is clear that using whole, unprocessed rapeseed is not a viable option but the other two rapeseed lipid forms reduced SFA (notably palmitic acid) and increased *cis*-MUFA (notably oleic acid).

**Table 2:** Effect of form of rapeseed lipids in the diet on milk fatty acid composition (g/100g fatty acids). (from [26])

Fatty acid	Treatment^1^				SEM^2^	*P^3^*
	Control	RO	WR	MR		
Σ ≤ 14:0	22.6^a^	18.2^c^	26.2^b^	21.3^a^	0.53	**
16:0	34.5^a^	19.8^c^	31.1^b^	21.6^c^	0.64	***
18:0	9.8^a^	14.6^b^	10.8^a^	15.5^b^	0.32	***
18:1 *trans* total	4.1^a^	10.0^c^	3.2^a^	6.4^b^	0.40	***
18:1 *cis* total	20.8^a^	26.9^b^	18.9^a^	25.6^b^	0.67	***
Σ saturates	69.6^a^	55.6^c^	71.7^a^	61.5^b^	0.86	***
Σ *cis* MUFA	22.7^a^	29.2^b^	21.4^a^	27.7^b^	0.75	***
Σ *trans* MUFA	4.4^a^	10.5^c^	3.5^a^	6.8^b^	0.40	***
*n*-6:*n*-3 PUFA	4.2	2.4	1.6	5.3	1.32	NS

^1^ Diets containing none (control) or rape lipid in the form of rapeseed oil (RO), whole rapeseeds (WR) or milled rapeseeds (MR). ^2^ Standard error of the mean for *n* = 16 measurements, 6 error degrees of freedom. ^3^ **P < 0.01 and ***P < 0.001, NS = not significant (P > 0.05). ^abc^ Means within row not sharing common roman superscripts differ significantly (*P* < 0.05).

## 3. Long Chain n-3 Fatty Acids and Chronic Disease

In the 1960’s and 70’s a number of studies demonstrated that consumption of fish was associated with a reduced risk of CHD in the Greenland Eskimos, despite an overall diet rich in fat [27,28]. This work led to the concept that the long chain (LC, carbon chain length ≥20) *n*-3 PUFA, in particular EPA (C20:5) and DHA (C22:6) typically found in marine foods were responsible for the observed cardioprotective effects. More recently, beneficial effects have been widely reported and include anti-atherogenic, anti-thrombotic and anti-inflammatory effects and overall, increased intakes leading to reduced risk of CHD (for a review, see [29]). 

Evidence is also accumulating that the intake of EPA and DHA may influence cognitive function in the elderly. The Zutphen Elderly Study examined fish consumption in 210 males, aged 70–89 y in 1990, along with estimates of cognitive function in 1990 and 1995 [6]. A significant (P < 0.01) positive linear trend was seen for the relationship between EPA+DHA intake and cognitive ability with a mean difference in intake of about 380 mg/d being associated with a 1.1-point difference in cognition. This study [6] concluded that moderate intakes of EPA+DHA may delay the decline in cognitive function in elderly men. Recent studies have however not shown an association between EPA/DHA intake and long term dementia risk [30,31]. 

The essential dietary component α-linolenic acid (C18:3 n-3, ALNA) can in theory be desaturated and elongated to EPA and DHA, but recent studies and reviews [32,33,34] have concluded that whilst the principal role of ALNA is indeed as precursor for EPA/DHA, the efficiency of conversion of ALNA to EPA is very low, especially in men, and that further transformation to DHA is often minimal. Indeed it was concluded that ALNA is probably a quite limited source of EPA/DHA in humans [33] which strongly suggests that these fatty acids should now be regarded as dietary essential. This is supported by the systematic review of Wang *et al*. [35] which concluded that increased consumption of n-3 fatty acids from fish or fish oil supplements, but not from ALNA, reduces the rates of all cause mortality, cardiac and sudden death and possibly stroke.

Givens and Gibbs [36] reviewed the current recommended intakes of EPA+DHA. Where such recommendations are made, typically they are in the region of 500 mg/d although there is evidence of benefits from considerably higher intakes [29]. A recent study into the establishment of a dietary reference intake for EPA+DHA also concluded that the most international recommendations are ~500 mg/d [34] although interestingly, the very recent scientific opinion from the European Food Safety Authority proposed a labelling reference intake for EPA+DHA of only 250 mg/d [37]. In the UK the target intake is 450 mg/d which is consistent with the consumption of two portions of fish per week, one of which is oil-rich. No consideration has yet been given to dietary recommendations in relation to brain function due to the relative paucity of data in this area.

### 3.1. Current Intakes of LC-n-3 Fatty Acids

The evidence relating to current intakes of EPA and DHA in various parts of the world has also been reviewed [36]. The data are summarised in Table 3. Some of the variability in estimated mean intake is likely to be due to the use of different food consumption surveys which suggest different levels of consumption of the key food types. Of note are the recommendations of SACN/COT [29] that canned tuna should be excluded from the oil-rich fish food category which makes it possible that some of the studies substantially overestimated EPA+DHA intake. It is also very important to realise that estimates of mean intake may not be very helpful. For example, in the UK, it was found that only about 27% of the adult population consume any oil-rich fish [29] and thus for the vast majority of the adult population the daily intake will probably at best be about 100 mg. A similar effect was seen in Belgian women [38]. Notably, in non-consumers of oil- rich fish in the UK, about half of the estimated 100 mg/d intake was provided by animal-derived foods, poultry meat in particular [39]. It is likely that much of the LC n-3 fatty acids found in poultry meat from birds which did not have fish oil in their diets is due to the diet containing fishmeal which contains some residual fish oil. In 2004 some 48000t of fish meal (25 % of total use) was used in the UK for poultry diets [40] although this has declined considerably since. Overall, it is clear that in many areas and central Europe in particular, intake of EPA+DHA is considerably sub-optimal and this may be a key public health nutrition issue.

**Table 3:** Recent estimated daily intakes of EPA + DHA in various countries (from [36]).

Country	Details	Intake of EPA + DHA(mg/person/d)
UK	Adults, 19-64 years, mean	244
UK	Females, 19-24 years, mean	109
Belgium	Females, 18-39 years, mean	209
Belgium	Females, 18-39 years, median	50
Belgium	Children 4-6.5 years, mean	75
France	Women 45-63 years	344
Australia	Adults	143
N. America	Adults	200
Central Europe	Adults	250
Northern Europe	Adults	590
Japan	Adults	950

### 3.2 Enriching Animal-Derived Foods with of EPA and DHA

Many studies have examined increasing the EPA and DHA concentration in animal-derived foods as a means of increasing daily intake (see for e.g. reviews [41,42]) although few have connected the potential for enrichment with current and projected patterns of food consumption. Assuming that consumption of enriched foods would be the same as that of normal foods, the potential for enrichment of a wide range of animal-derived foods has been calculated and how these may contribute to additional EPA+DHA intake in the UK [38]. The findings for milk and milk products, meat and eggs are summarised in Table 4. 

**Table 4:** Potential mean intakes of EPA and DHA by adults in the UK from enriched animal-derived foods (from [39]).

Food	Intake (g/person/week)^1^	Concentration^2^ (mg/g) of		Intake of EPA+DHA (mg/person/d)
		EPA	DHA	
*Milk products*				
Whole milk	337	0.106	0.141	11.9
Semi-skimmed milk	877	0.045	0.060	13.2
Skimmed milk	215	0.008	0.011	0.57
Cream	12	1.064	1.406	4.27
Other milk	42	0.080	0.105	1.12
Cottage cheese	9	0.104	0.137	0.31
Other cheese	98	0.745	0.984	24.2
Butter	22	2.181	2.882	16.0
Total milk products:				71.5
*Meat*				
Beef and veal	249	0.24	0.053	10.4
Sheep meat	51	0.82	0.97	13.0
Pork	63	0.13	0.167	2.67
Bacon and ham	105	0.072	0.093	2.47
Poultry	374	0.60	0.80	74.8
Sausages^3^	68	0.012	0.015	0.26
Other products^3^	216	0.036	0.006	1.70
Total meat products:				105.4
*Eggs*	194	0.06	1.90	54.3
Total intake:				231

This shows that enrichment of animal-derived foods has the potential to provide a daily intake of EPA+DHA of about 230 mg/person/day with poultry meat providing the largest potential intake (74 mg). The contribution of poultry meat is a result of it being consumed in large quantities and its amenability to enrichment. Other useful contributions could be provided by eggs and full fat cheese although the contributions from liquid milk and other meats are likely to be very modest based on current food consumption data. 

### 3.3 Enrichment of Poultry Meat

The concentrations of EPA and DHA in poultry meat can in theory be relatively easily increased by including fish oil in the diet of the birds. This can result in nutritionally meaningful increased intakes of these fatty acids by people who consume the meat. This topic has been reviewed recently [43]. One thing this review identified was the lack of data relating to the relative ability to enrich the meat of modern genotypes of broiler chickens and turkeys. Subsequently a study on this was done to determine the relative increases in the EPA and DHA in edible tissues to increased concentrations of fish oil in the diet [44]. Some key findings for skinless white chicken meat are shown in Table 5. 

**Table 5:** Effect of fish oil in the diet and breed of broiler chicken on the mean EPA and DHA concentration (mg/100g meat) in white chicken meat (from [44]).

	Control^1^		Lofish		Hifish		P	
Fatty acid	Ross^2^	Cobb	Ross	Cobb	Ross	Cobb	Breed	Diet
EPA	7.5	6.9	17.4	20.0	27.2	30.8	NS	<0.001
DHA	39.6	38.6	54.9	64.3	118	126	NS	<0.001

^1^ Diets contained fish oil at Contol 0, Lofish 20 and Hifish 40 g/kg diet. ^2^ Breed of birds used, Ross 308 and Cobb 500

Overall, the results showed that, in modern broiler genotypes, there was no significant difference in the efficiency with which EPA and DHA were incorporated into edible tissue. There was also little evidence to suggest that there is any inherent difference between broilers and turkeys in their ability to incorporate EPA and DHA into their edible tissues, except perhaps for EPA in white meat. As shown in Table 5, the diets containing 40 g fish oil per kg gave rise to white chicken meat containing about 140 to 160 mg EPA+DHA per 100 g which has the potential to make a real contribution to dietary EPA+DHA intake.

There are some potential drawbacks to enriching poultry meat with EPA and DHA since the meat will potentially have reduced oxidative stability and organoleptic qualities. Current work in this laboratory indicates that such problems can be minimised by use of additional vitamin E in the diet (~200 mg/kg) of the bird although for commercial application this will need to be coupled with appropriate meat processing, packaging and storage strategies.

## 4. Conclusions

Foods derived from animals are an important source of nutrients in the diet. However, certain aspects of some of these foods, SFA in particular, have led to concerns about the contribution of these foods to increased risk of CVD and other conditions. The fatty acid composition of various animal derived foods is not constant and can, in many cases, be enhanced by animal nutrition. The future role of animal nutrition in creating foods closer to the optimum composition for long term human health will be increasingly important. However, certain animal derived foods contain compounds which actively promote long term health and research is urgently required to fully characterise these effects and to determine how the levels in natural foods may be enhanced. 

## Acknowledgments

This paper is based on a presentation given at the International Conference FOOD-OMICS, University of Bologna, Cesena, 28-29 May, 2009 and was supported by LIPGENE, an Integrated Project within the EU funded Sixth Framework Research programme (www.ucd.ie/lipgene) and by Feed for Health, COST Action FA 0802 (www.feedforhealth.org).
